# Rehabilitation of Worn Dentition with Direct Resin Composite Restorations: A Case Report

**DOI:** 10.3390/dj10040051

**Published:** 2022-03-23

**Authors:** Marta Blasi Beriain, Giovanni Tommaso Rocca, Leonardo Franchini, Didier Dietschi, Carlo Massimo Saratti

**Affiliations:** 1 Faculty of Dentistry, University of Geneva, Rue Lombard 19, 1205 Geneva, Switzerland; giovanni.rocca@unige.ch (G.T.R.); didier.dietschi@unige.ch (D.D.); carlo.saratti@unige.ch (C.M.S.); 2 Private Practice, CDT, 50137 Florence, Italy; franchini.leonardo@gmail.com

**Keywords:** full mouth rehabilitation, tooth wear, resin composite

## Abstract

The incidence of tooth wear has steadily increased in all Western populations during the past decades. A through-care strategy, extendable for a lifetime, has become crucial to prevent the extensive loss of sound dental structure and to make an eventual retreatment affordable in the long term. An interceptive treatment using resin composite materials and no-preparation approaches meets these requirements. Moreover, continual developments in digital dentistry makes possible to predict the treatment plan for the restorative rehabilitation of the mouth. The availability of digital resources allows clinicians to increase predictability for excellent esthetics and good functional results. This article provides a step-by-step description of a full-mouth additive rehabilitation achieved by employing digital workflows and direct resin composite restorations. A comprehensive functional and esthetic evaluation of the treatment is proposed and discussed.

## 1. Introduction

Tooth wear is a concerning problem with a rising rate of occurrence, as the prevalence of dental caries is decreasing and teeth have longer life [1]. The level of wear seen in elderly patients might be expected and acceptable, while similar levels in young patients can be identified as pathological [2].

Pathological tooth wear is defined as “atypical to the age of the patient, causing pain or discomfort, functional problems, or deteriorations in aesthetic appearance, which, if progressing, may give rise to undesirable complications of increasing complexity” [3]. Its etiology can be mechanical (attrition, abrasion, and abfraction) or chemical (erosion), and both etiologies can be caused by intrinsic or extrinsic factors. However, most of the time it is difficult to identify the main cause of the problem, as many patients present combined etiologies [4].

Dentinal hyper sensibility, a consequence of aggressive dental erosion, has been reported as a major possible issue for patients. In fact, when the progression of dental sound tissue loss surpasses the dentin-pulp complex reparative capacity, it might provoke toothache, pulpal inflammation, pulp necrosis, and periapical lesions of some dental elements. However, the major cause of patients’ complaints is the degradation of the esthetic appearance of the smile, with dentine exposure, chipping and fracture of incisal edges, loss of micro- and macro-anatomy, and tooth shortening [1].

Dental erosion is the loss of dental sound substance as a consequence of intrinsic (acid reflux, bulimia nervosa) or extrinsic (food, beverages) acid attacks. Such a phenomenon may possibly be diagnosed years after the beginning of the erosion activity, which occurs either by hydrogen ion attack or by the action of chelating anions that dissolves the integrity of enamel crystals [4]. The development and intensity of dental erosion will depend on the interplay with other risk factors such as biological factors (quality of saliva, tooth structure, soft tissues architecture), individual behavioral factors (unhealthy lifestyles, healthy lifestyles with consumption of sport drinks, nutritional habits), systemic health, awareness of the risk factors and socioeconomic status [5]. The assessment of preventive measurements is based on the identification of risk factors, and should be thoroughly and routinely investigated in the clinical setting. Discussing drinking and eating habits, giving dietary advice, or referring the patient to a gastroenterologist if acid reflux is present will help in the management of these risks. It is recommended that a patient reduce the amount and frequency of intake of acidic drinks, changing drinking habits (such as “swishing” or “sipping,” which increase the duration of the contact between the dietary acid and dental surface), and rinse their mouth with water after the consumption of the acidic substance [6].

Minimally invasive and adhesive dentistry strategies of rehabilitation are now preferred when treating a worn dentition. This is especially important for young patients, for whom a comprehensive, lifelong approach is essential [7]. Such techniques are beneficial for increasing the vertical dimension of occlusion to maximize the preservation of sound dental tissue (VDO). This method provides multiples advantages, including harmonizing dentofacial esthetics, providing adequate space for the planned restorations, and improving incisal and occlusal relationships and guidance [8].

Treatment approaches should integrate minimal invasive concepts and prophylactic care, especially when young patients are involved [9]. Conventional preparation with full-coverage ceramic crowns requires tooth reduction of from 40 to 70%, while minimally invasive protocols preserve dental structure and avoid elective endodontic therapy [10]. Due to the improvement in adhesive techniques and resin composite materials, the indications for full coverage crowns have undergone a major downsizing [11].

The development of digital technologies is helpful in measuring precisely the amount of dental hard tissue loss, and consequently in increasing the predictability of the rehabilitation [12]. The continuous upgrades of functions available and the improvement of the performances of digital devices allows an increased spectrum of action for clinicians, permitting them to undertake new and more integrated restorative workflows [13,14,15].

The goal of this clinical report is to present a full mouth rehabilitation performed with direct resin composites using fully digital planning.

## 2. Clinical Report

**Figure 1:**
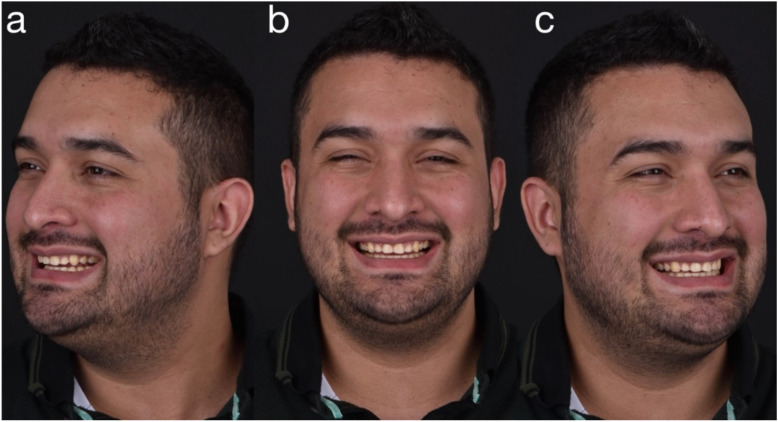
Extraoral facial smiling pictures (**a**) left, (**b**) frontal, (**c**) right.

**Figure 2:**
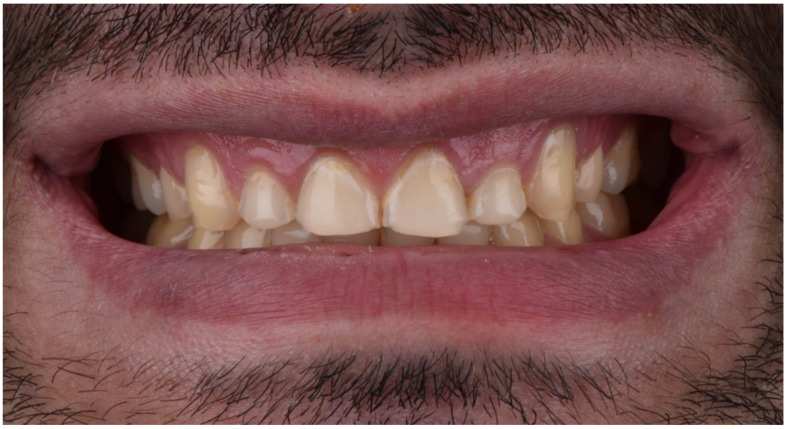
Extraoral smile picture (front).

**Figure 3:**
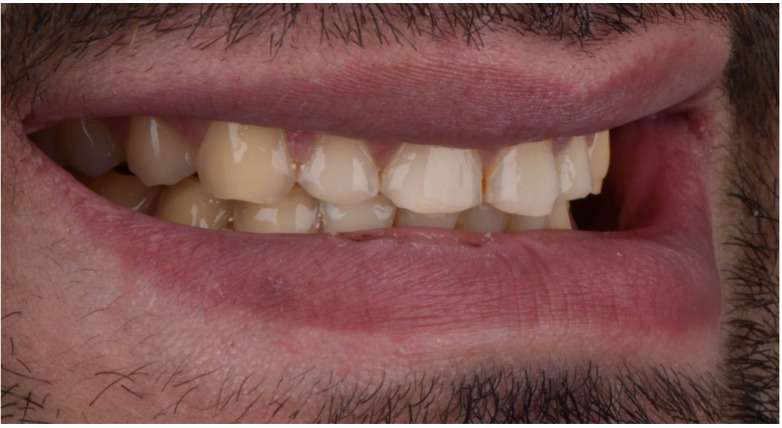
Extraoral smile picture (right).

**Figure 4:**
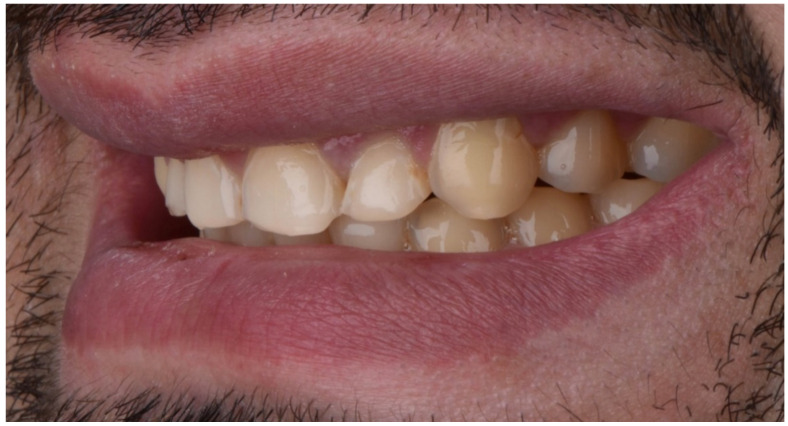
Extraoral smile picture (left).

**Figure 5:**
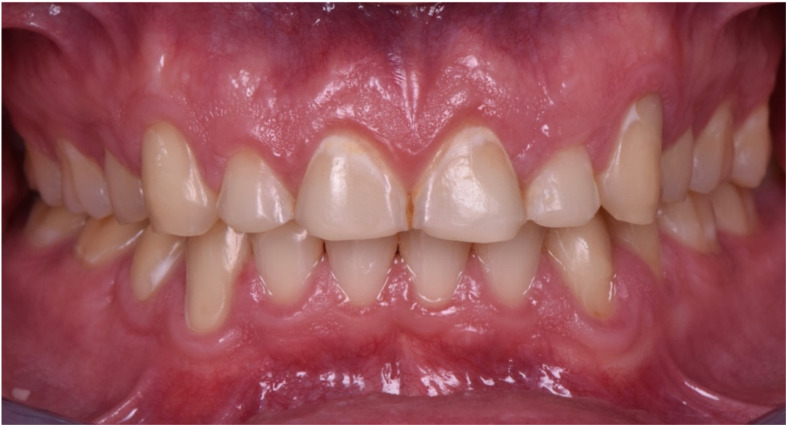
Intraoral picture (front).

**Figure 6:**
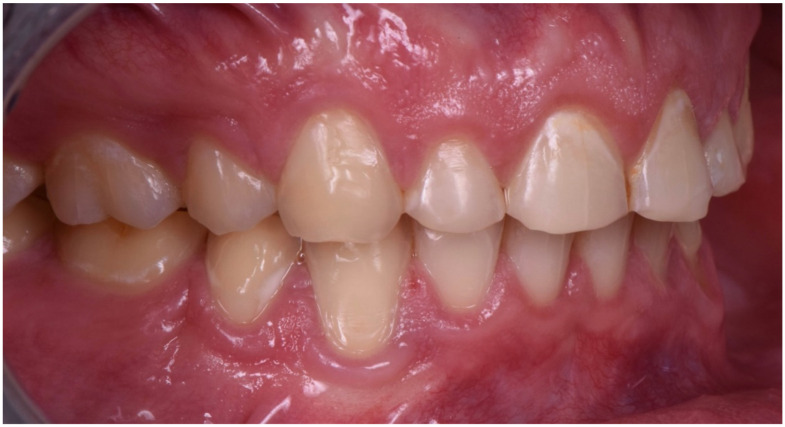
Intraoral picture (right).

**Figure 7:**
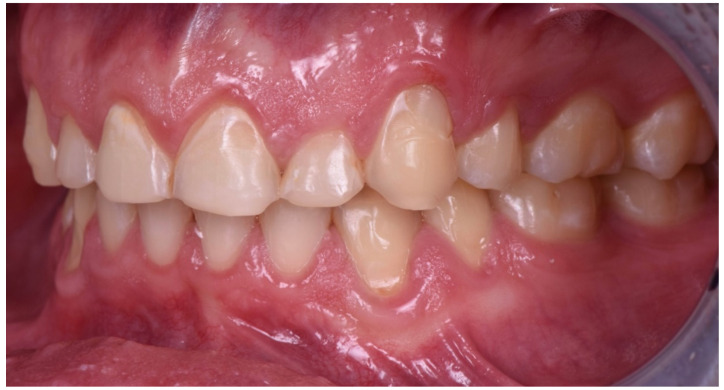
Intraoral picture (left).

**Figure 8:**
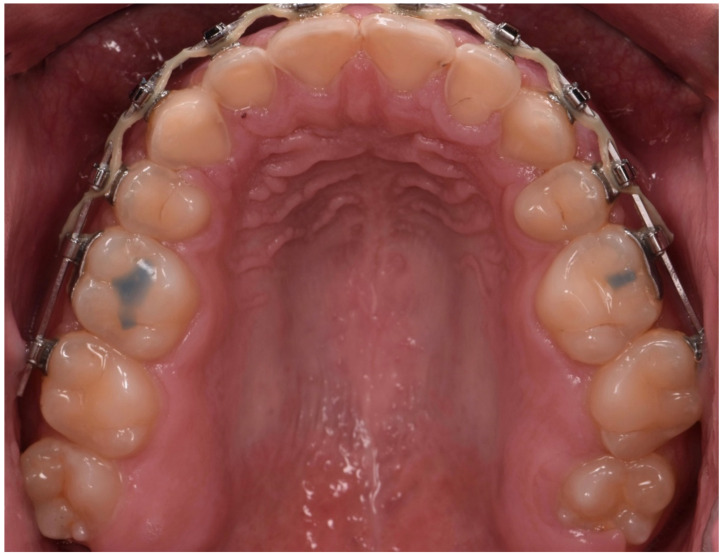
Intraoral occlusal picture (upper arch).

**Figure 9:**
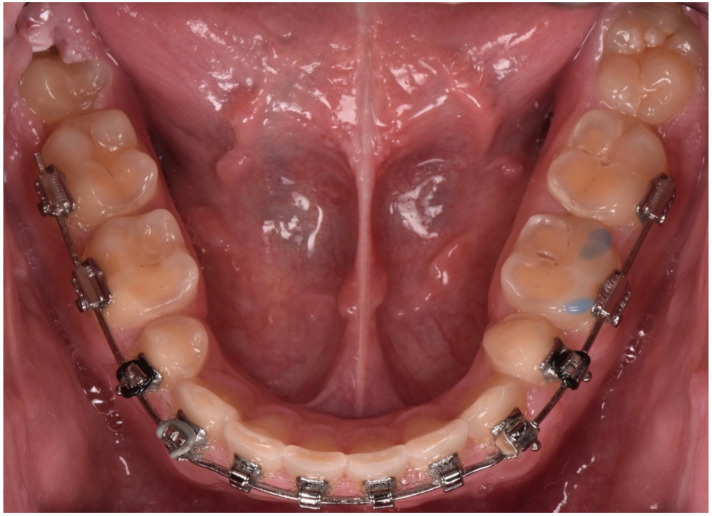
Intraoral occlusal picture (lower arch).

A 28-year-old male patient presented to the dental clinic at the University of Geneva, having been referred to restore his worn dentition after completing orthodontic treatment. An intraoral clinical examination revealed excessive tooth wear, which had led to the exposure of dentinal tissue. An initial documentation of the patient was used to create the treatment plan and as a tool for designing a future smile. (Figure 1, Figure 2, Figure 3, Figure 4, Figure 5, Figure 6, Figure 7, Figure 8 and Figure 9). The patient reported a prediabetic condition, and after a detailed discussion, he revealed a high rate of consumption of carbonated beverages. He also noted a bruxism habit. The Tooth Wear Evaluation System (TWES) index score was used to obtain a precise diagnosis of the patient’s tooth wear, which was classified according to TWES 2.0 as “generalized moderate tooth wear and localized severe pathological tooth wear, mainly chemical and partially mechanical” [16].

The functional evaluation of TMJ was initiated with the screening questionnaire from the Diagnostic Criteria for Temporomandibular Disorders (DC/TMD) [17]. The questionnaire resulted in negative and dysfunctional pathologies of the stomatognathic system being excluded. In fact, the patient did not refer to pain at extrusive movements, nor were there any clicking, tripping noises, or crepitus found. Functional analysis was completed using the Kinesiography system (BIO-key; Bioket, San Benedetto del Tronto, Italy), which helped us analyze the jaw cinematically during opening protrusive and lateral movements [18,19].

### 2.1. Planning Phase

First, a motivational interview was organized with the clear goal of reducing the patient’s intake of carbonated beverages in order to stabilize the erosive process. The patient then received professional dental hygiene therapy, including oral hygiene technique instructions. Virtual files of the initial situation were recorded in the standard tessellation (STL) format by the use of a digital intraoral scan (CEREC Primescan, Sirona, York, PA, USA). A facial scan was also recorded using a specific application (Face App, Bellus 3D, San Jose, CA, USA) with a personal smart phone.

The intermaxillary relationship of rehabilitation was established following the myocentric position concept and according to the minimal space needed to restore the loss of tooth structure. The myocentric position is a position referring to the muscles established along the neuromuscular trajectory. In this position, muscles are simultaneously at their resting length and in balanced tonus in relation to one another [20].

Mandibular muscles were deprogrammed using cotton rolls (8 mm thick) between the posterior teeth (from canine to second molar). The patient was asked to stay at rest, perform movements of the jaw, and swallow several times for a period of 5 min. The bite registration was done on hard silicone (Stonebite VPS, Dreve Dentamid GmbH, Unna, Germany) after cotton rolls were removed and the patient was asked to close until the minimal space required for restorations was commensurate with a good anterior overjet and overbite. Initial evaluation was done subjectively during the clinical session. A validation of this step was obtained through subsequent analysis of digital models with the wax-up in the laboratory and measurement of spaces obtained with the VDO augmentation. The hard silicone was not removed until an STL file was created registering the intermaxillary relationship, in order to transmit to the laboratory the position and space available for the restorations.

**Figure 10:**
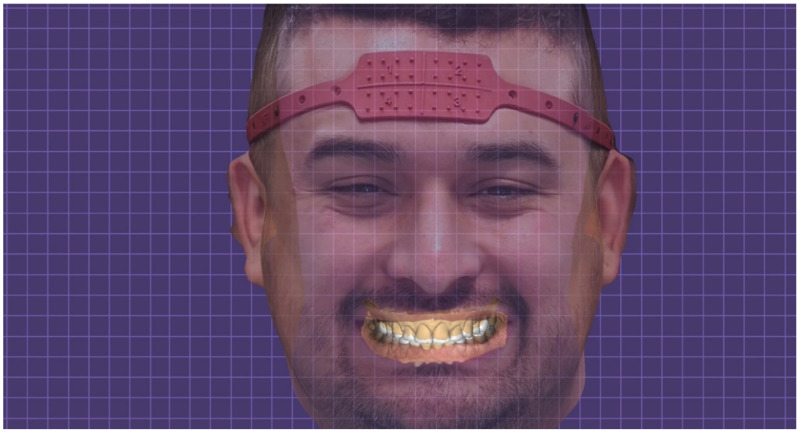
STL Digital wax-up integrated with 3D facial scan.

**Figure 11:**
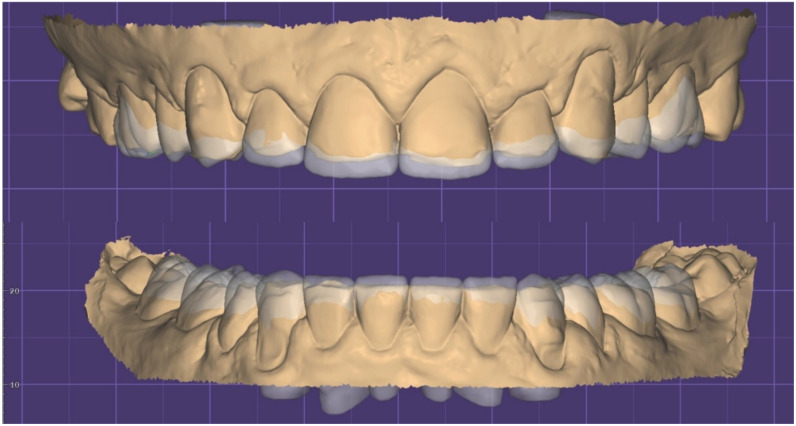
STL Digital wax up (front).

**Figure 12:**
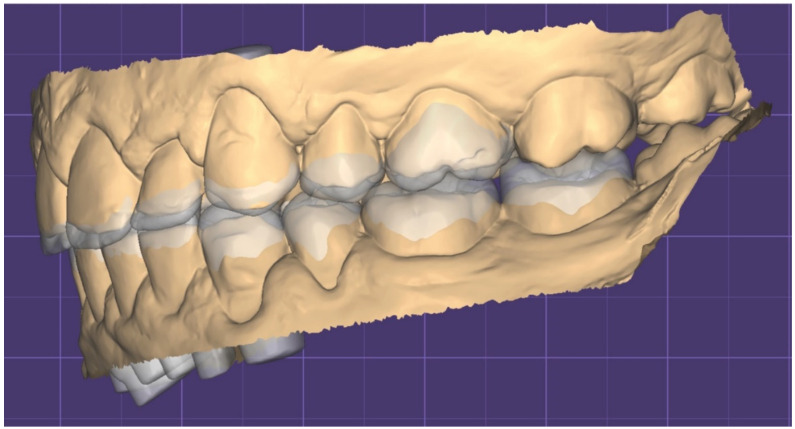
STL Digital wax up (left).

**Figure 13:**
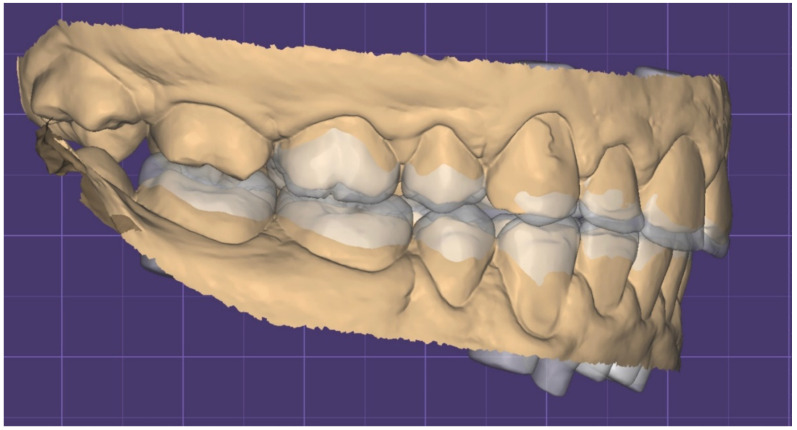
STL Digital wax up (left).

The extraoral and intraoral pictures and the STL files were sent to a laboratory technician along with the facial scan. A digital wax-up was created to plan the rehabilitation project, which was integrated with a 3D scan of the patient’s face (Figure 10, Figure 11, Figure 12 and Figure 13).

**Figure 14:**
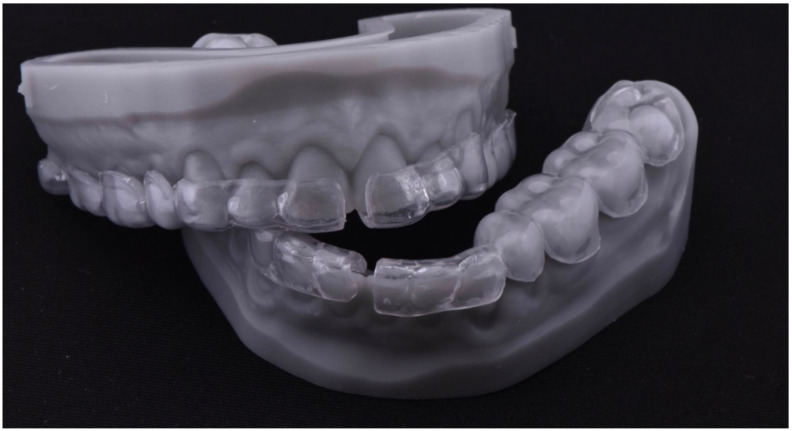
Heat forming hard trays.

**Figure 15:**
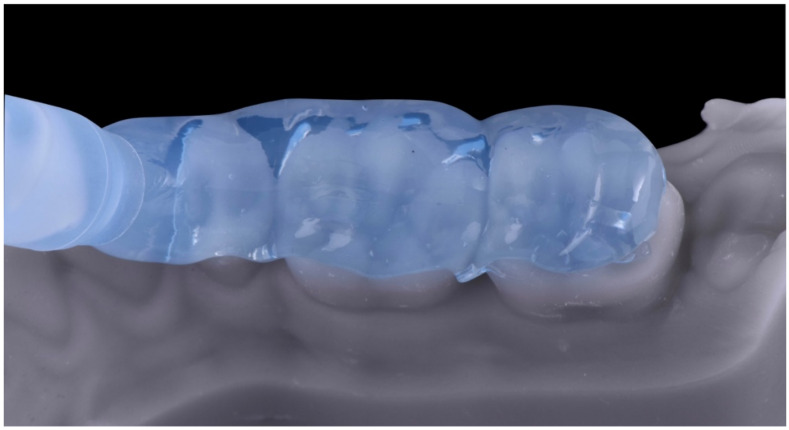
Transparent silicone (Memosil).

**Figure 16:**
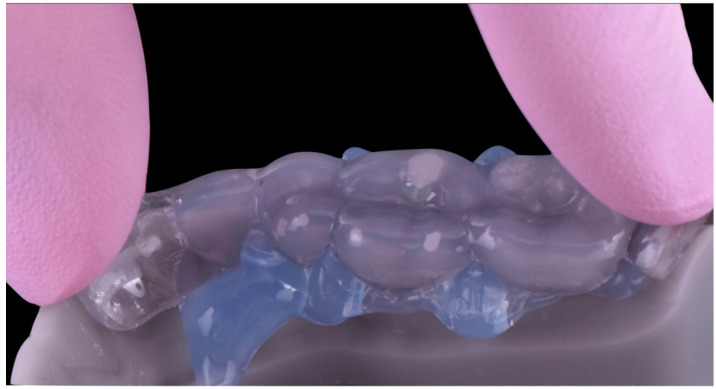
Heat-forming hard trays relined with transparent silicone.

**Figure 17:**
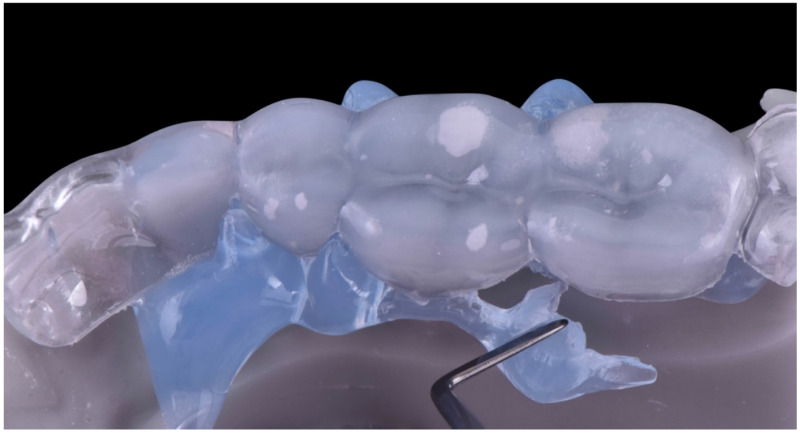
Removal of transparent silicon excesses.

The digital version of the full-molding technique was used to guide the clinical restorative phase [21]. After finishing the project of the rehabilitation, it was 3D-printed in high definition. A 2-mm–thick hard tray (Erkodur; Erkodent, Pfalzgrafenweiler, Germany) was then fabricated by heat forming on the 3D-printed models (Figure 14). It was separated into a left and right part, maintaining double stabilization points (mesial and distal), which were localized on the surface of the second molar and anterior teeth. Internal sandblasting of the trays was done with 50 μm alumina particles for 10 s with a pressure of 4 bar, in order to provide surface irregularities that would enhance the mechanical bonding for the next step. The trays were relined with a clear silicone (Memosil 2; Heraeus, Hanau, Germany) directly on the printed project (Figure 15, Figure 16 and Figure 17).

### 2.2. Restorative Phase

**Figure 18:**
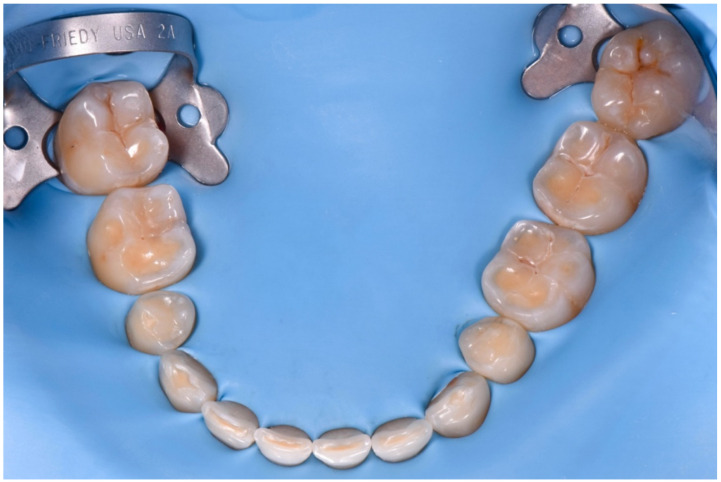
Full arch isolation with rubber dam.

**Figure 19:**
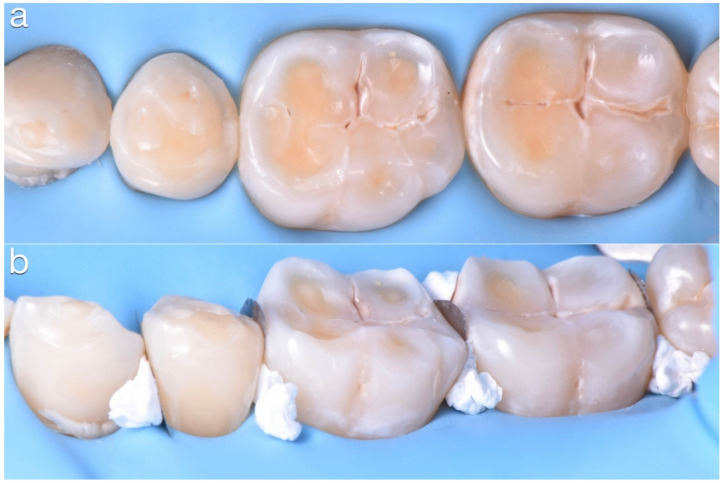
(**a**) Rubber dam isolation, (**b**) isolation of interproximal spaces with Teflon and customized matrix.

It was decided to rehabilitate the posterior teeth first to stabilize the occlusion. Each quadrant was treated separately. The mandibular arch was isolated with rubber dam (Figure 18) after the try-in of the tray to evaluate the precision, the accessibility to the contact points, and the mesial and distal stops. In order to avoid penetration of the composite, the interproximal spaces were filled with Teflon and a customized matrix (Figure 19).

**Figure 20:**
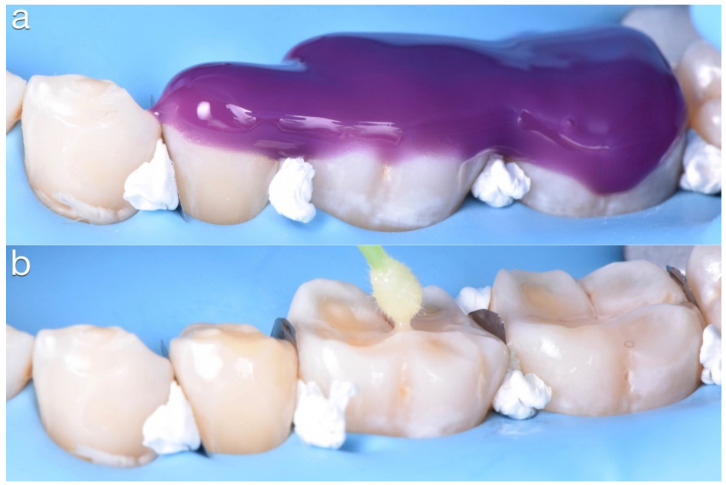
(**a**) Selective etching using 37% phosphoric acid for 30 s on enamel and 15 s on dentin, (**b**) Adhesive luting procedure.

No dental preparation was performed, except for a complete sandblasting of the occlusal surfaces (27 μm alumina powder) to clean the tooth surface and to remove the amorphous enamel domains, thus exposing the underlying prismatic structure, which has better adhesive conditions [22]. A selective etching using 37% phosphoric acid (Ultra-Etch; Ultradent, South Jordan, USA) for 30 s on enamel and 15 s on dentin was then done and a three-step etch-and-rinse adhesive system (OptiBond FL; Kerr, San Antonio, TX, USA) was applied and polymerized for 20 s (Figure 20).

**Figure 21:**
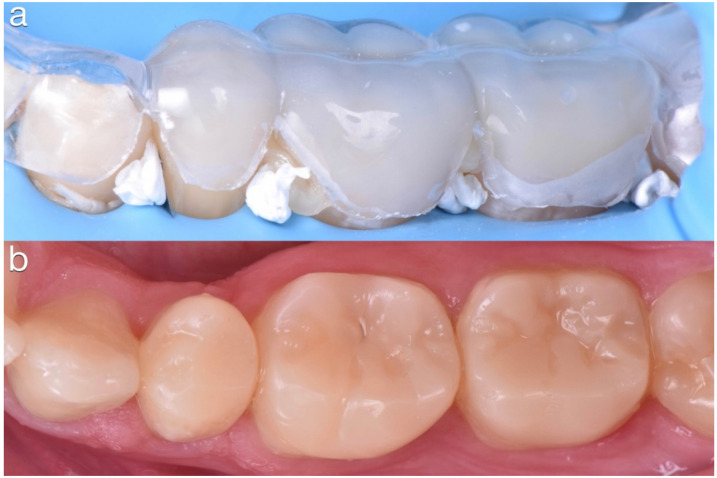
(**a**) Plastic tray filled with preheated hybrid composite and placed in the corresponding quadrant. (**b**) Final result.

A preheated hybrid composite resin (Inspiro Skin White, Edelweiss DR, Zug, Switzerland) was placed inside the plastic tray, positioned in the corresponding quadrant and pressed with gentle pressure until the tray fitted the mesial and distal stops (Figure 21). Before light curing, excesses were removed from the interproximal surfaces (as the tray was open until the contact point) and from the lingual and vestibular surfaces. Then, 20 s of light curing was performed on each element before the tray was removed, and 20 s was performed on every surface after the tray was removed. With the help of a metallic instrument (Elliot separator, Dentech, Farmington Hills, MI, USA), the interproximal surfaces were polished and remaining excesses were removed. Finishing was carried out with discs and fine diamond burs, and polishing was completed with rubber points. The same process was repeated in each posterior quadrant, leaving the patient an open anterior bite. At the end of the appointment, the occlusal contacts were checked, and occlusal stability was determined to exist, as every restored tooth presented one or more occlusal contacts.

**Figure 22:**
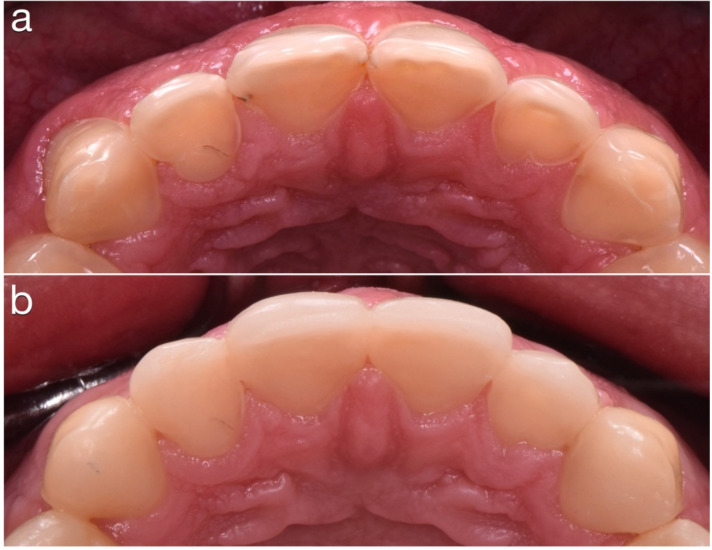
(**a**) Palatal surfaces before restorations were done. (**b**) Palatal direct composites.

**Figure 23:**
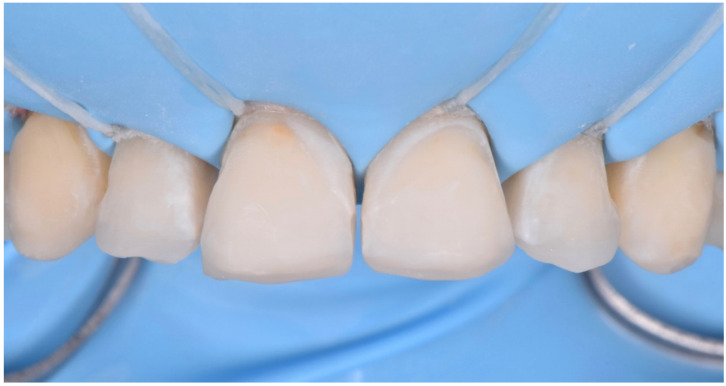
Rubber dam isolation with ligatures.

**Figure 24:**
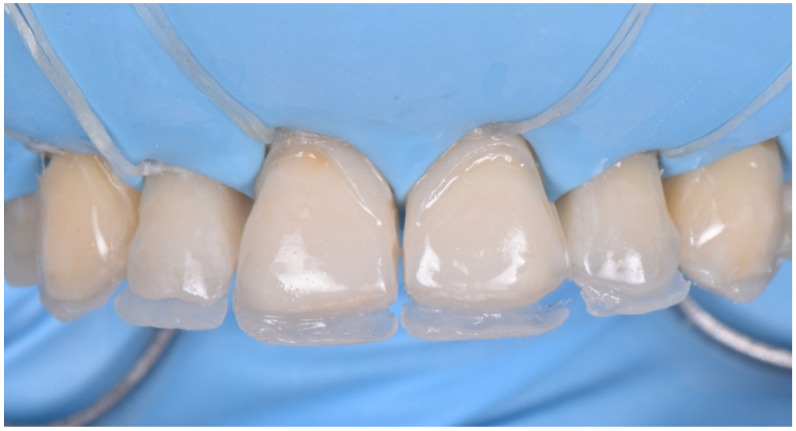
Enamel palatal shells following the “natural layering concept”.

**Figure 25:**
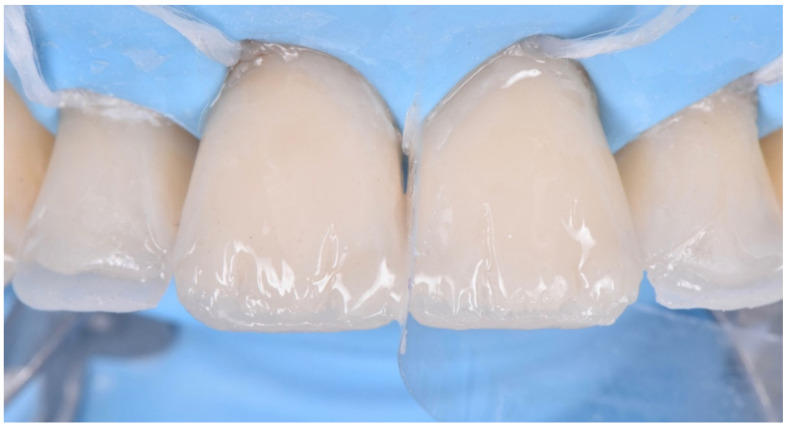
Dentin layer following the “natural layering concept”.

**Figure 26:**
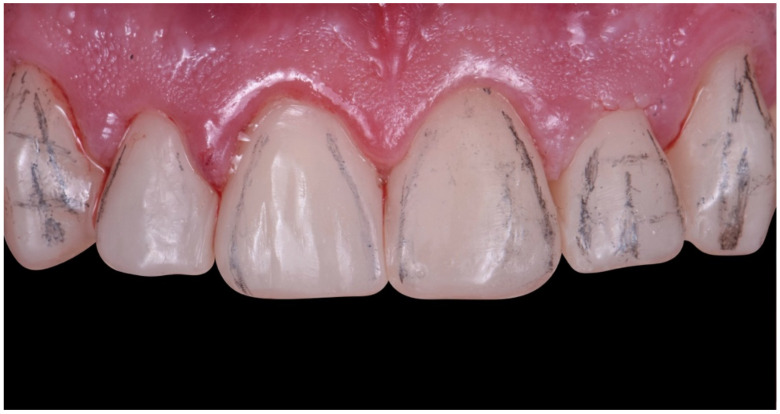
Finishing and polishing of the anterior composites.

After two weeks, the restoration of the palatal and vestibular anterior surfaces was done. After isolation of the operatory field with a rubber dam, a free-hand composite was placed on the palatal surfaces from teeth 1.3 to 2.3, following the same adhesive protocol used in the posterior quadrants (Figure 22). Subsequently, the esthetic buccal surfaces of the anterior restorations were restored. With the help of a silicone palatal key created on a printed digital projector, the length of the incisal margin was determined and built up with the same resin composite used for the posterior restorations (Inspiro, Skin White and Bi2; Edelweiss DR), following the cleaning of white spots present on the cervical third (Figure 23). The same adhesive protocol and material were applied in the antero-inferior sextant, using the “natural layering concept” to stratify different shades of composites (Figure 24 and Figure 25) [23] During the last 40 min of the session, detailed finishing and polishing procedures were performed to improve the outcome of primary and secondary anatomy, and get smooth and natural composite surfaces (Figure 26). The primary anatomy was corrected with finishing discs (abrasive medium-grift Sof-Lex Disc, 3M) and the secondary anatomy was completed with a red-ring diamond flame bur, followed by a pre-polisher rubber point (Identoflex minipoint, Kerr) and a diamond-impregnated rubber cup (Optrapol, Ivoclar Vivadent, Schaan, Liechtenstein). Restorations of lower incisors were performed following the same restorative protocol.

**Figure 27:**
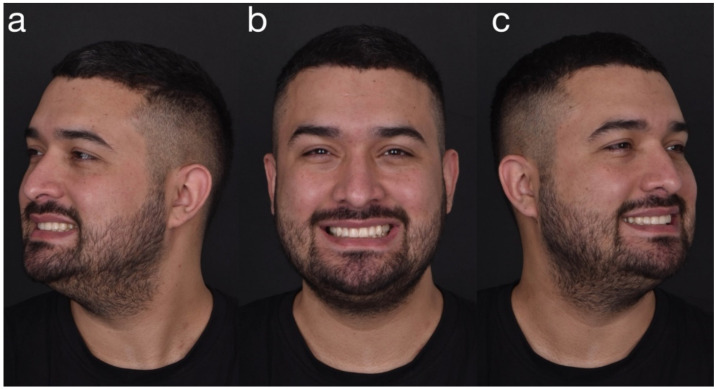
Final extraoral facial smiling pictures ((**a**) left, (**b**) frontal, (**c**) right).

**Figure 28:**
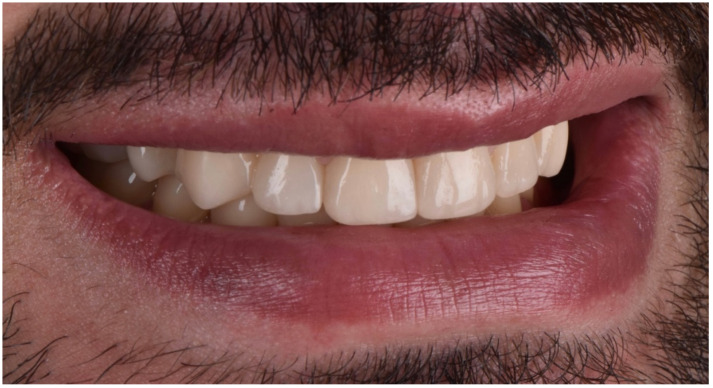
Final extraoral smile picture (right).

**Figure 29:**
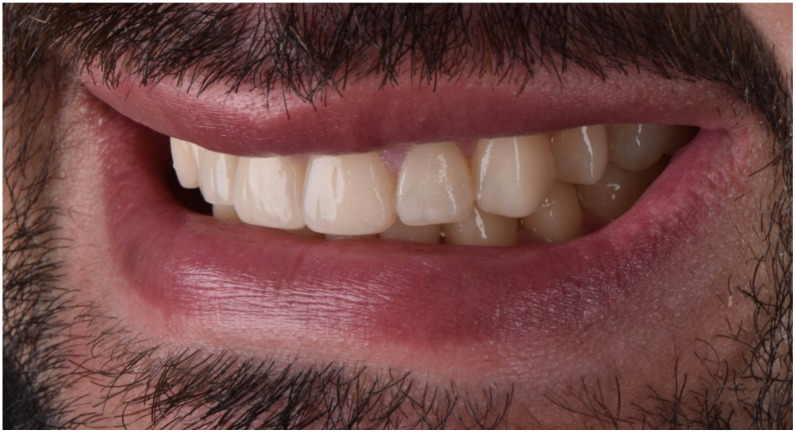
Final extraoral smile picture (left).

**Figure 30:**
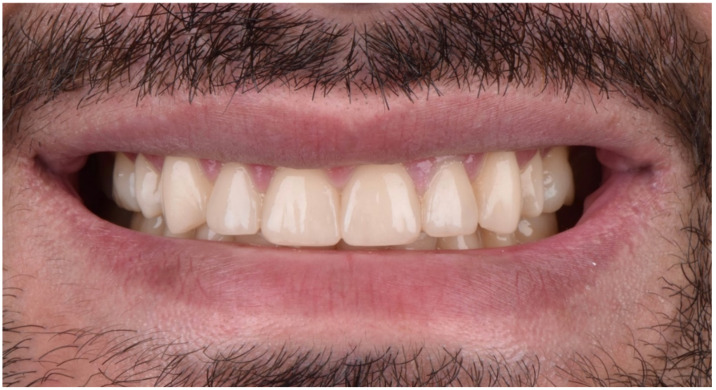
Final extraoral smile picture (front).

**Figure 31:**
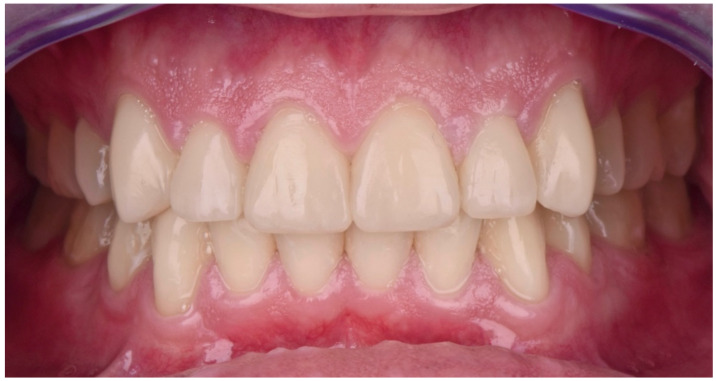
Final intraoral picture (front).

**Figure 32:**
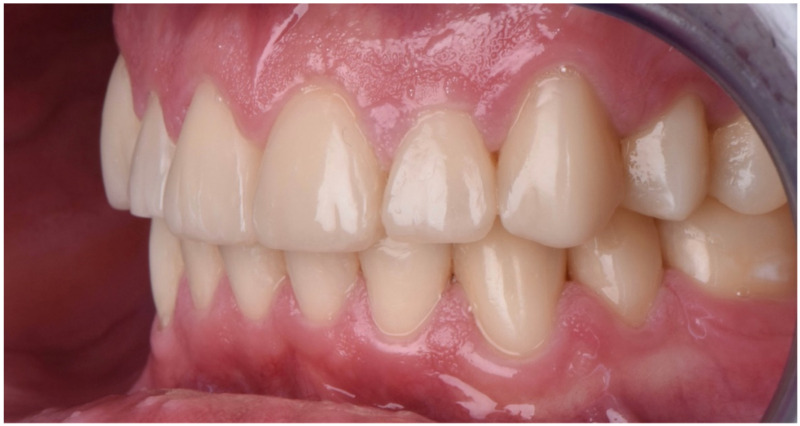
Final intraoral picture (left).

**Figure 33:**
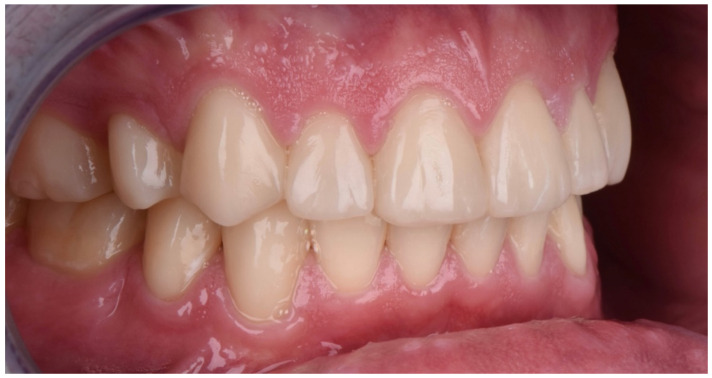
Final intraoral picture (right).

**Figure 34:**
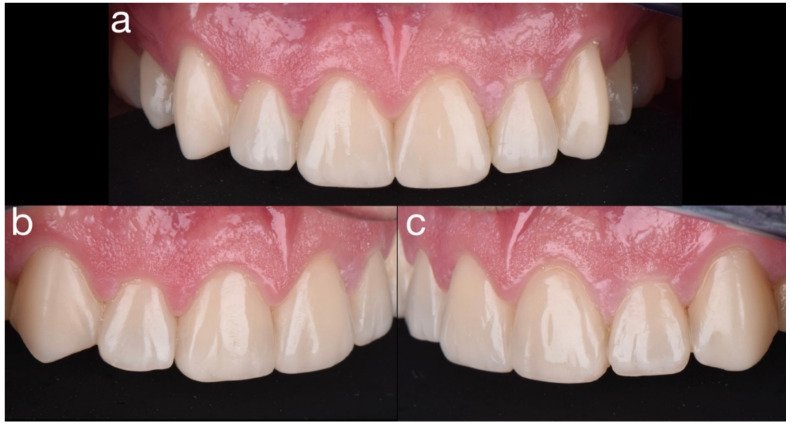
Final upper anterior restorations ((**a**) frontal, (**b**) right, (**c**) left).

**Figure 35:**
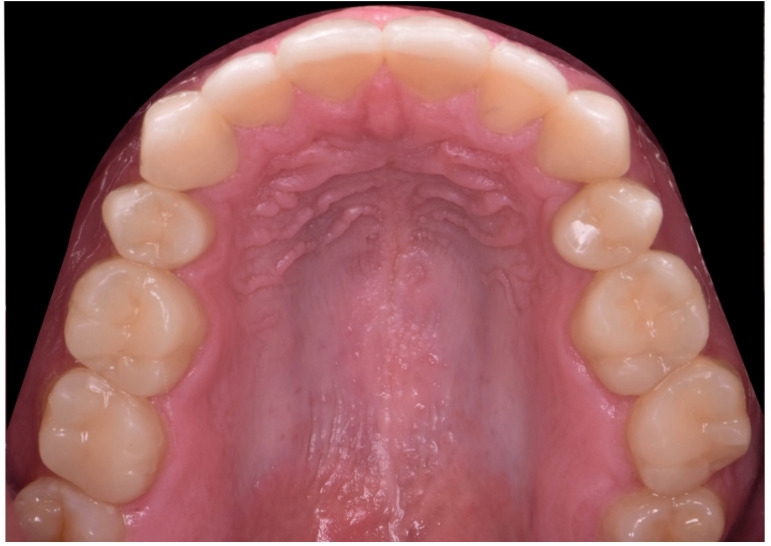
Final intraoral occlusal picture (upper arch).

**Figure 36:**
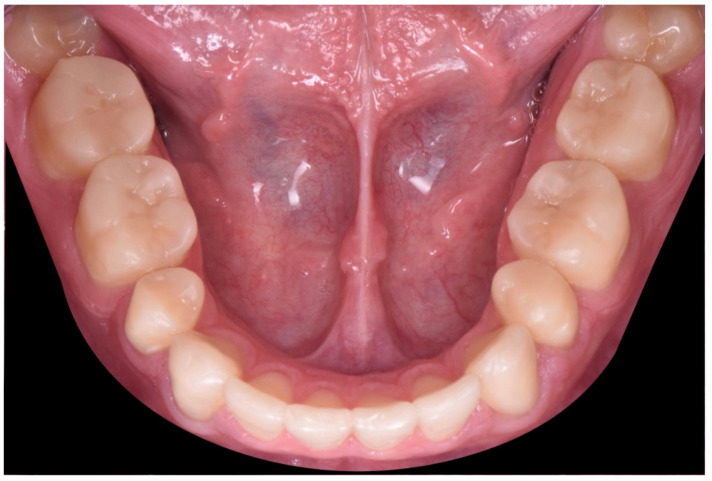
Final intraoral occlusal picture (lower arch).

**Figure 37:**
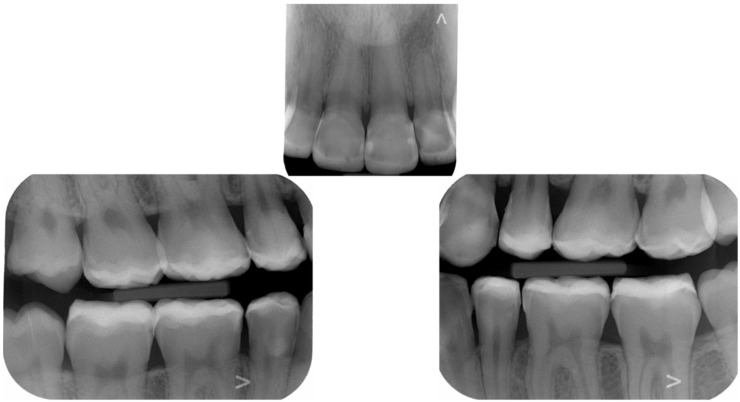
Final radiological documentation.

**Figure 38:**
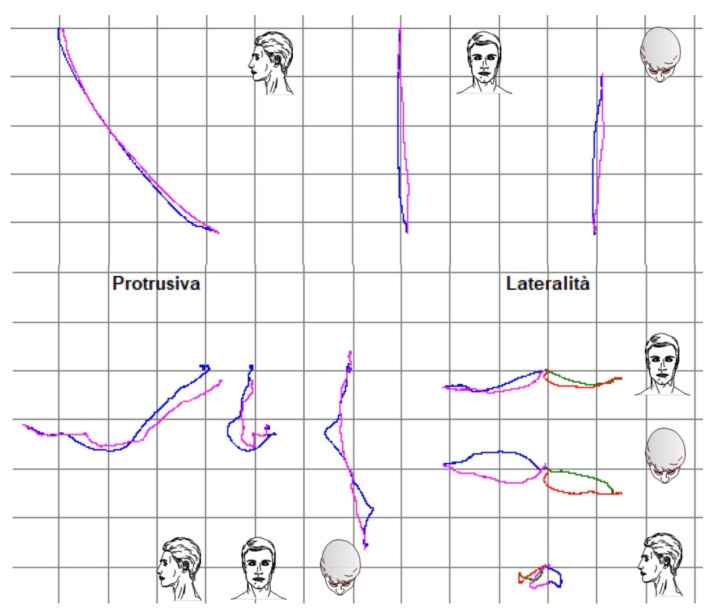
Comparison of kinesiograpic analysis before and after treatment.

**Figure 39:**
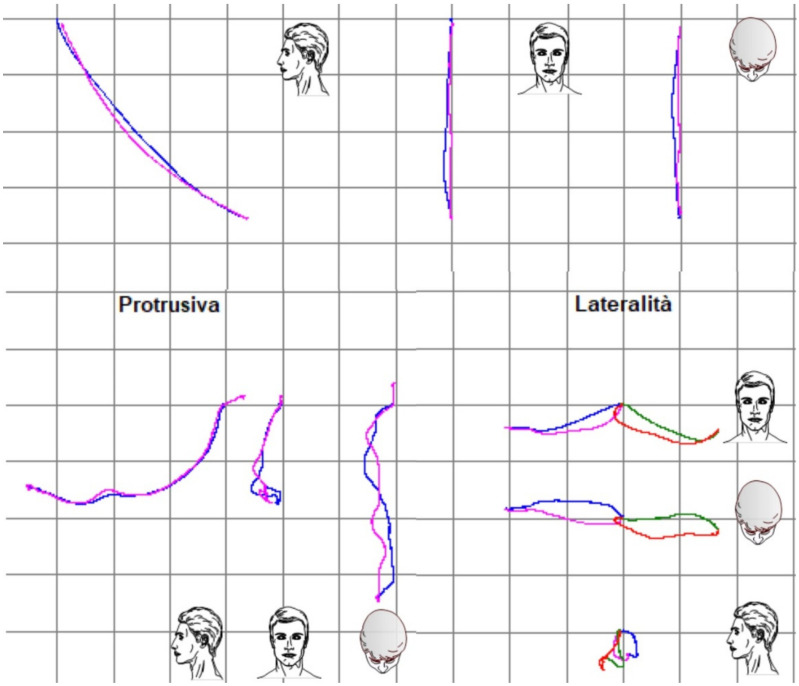
Comparison of kinesiograpic analysis before and after treatment.

The final post-treatment documentation with intraoral and extraoral pictures (Figure 27, Figure 28, Figure 29, Figure 30, Figure 31, Figure 32, Figure 33, Figure 34, Figure 35 and Figure 36) and X-ray (Figure 37) was done one month after the end of the treatment. Once treatment was completed, the patient was aware of the need for excellent oral hygiene, the importance of low consumption of a cariogenic and acidic diet, and the need to wear a rigid, heat-formed, 1.5 mm-thick maxillary protective night guard, which was given to the patient as a protective appliance. A final kinesiography was taken to record and compare with the initial analysis (Figure 38 and Figure 39).

## 3. Discussion

In this present patient, the primary focus of advice to manage the tooth wear was decreasing frequency of dietary acid intake. It was accomplished by showing images of eroded teeth and the use of his 3D teeth scans, which were helpful in motivating the patient and increasing his compliance. With the use of digital monitoring software and the scored level of tooth wear employed (TWES 2.0), it will be possible to monitor and record the severity of dental wear in the future [17].

Modification of the VDO has been one of the most controversial issues in restorative dentistry for a long time, but it can be successfully accomplished if proper diagnosis and treatment planning are performed [24]. The therapeutically designed VDO it is not an immutable reference point, but a dynamic dimension within a zone of physiological tolerance (comfort zone) [18]. Up to 5 mm is considered a predictable and safe procedure (any signs and symptoms tend to be self-limiting and to resolve within two weeks) [25]. As mentioned previously, muscular deprogramming was carried out in this patient using cotton rolls between the lower and upper teeth, in order to obtain a new intermaxillary relationship determined by the muscle contraction [20,26]. To determine the VDO, several criteria were established: the patient’s esthetic satisfaction, biomechanical, and functional needs and minimal thickness of the restoration for the sake of minimally invasive approach [8]. As the determined VDO modification was around 2–3 mm, a test for its validation was estimated to be unnecessary. In fact, the patient adapted to the new occlusal conditions in less than one week. Moreover, the use of kinesiography showed that jaw mobility was almost unchanged before and after treatment. In fact, precise evaluation and measurement of jaw dynamics can be helpful in the functional assessment of a full-mouth rehabilitation [19].

After the amount of increase of VDO was determined, a virtual 3D diagnostics wax-up between both arches was accomplished. The facial scan enabled the transferring of the position of the maxillary jaw to be aligned with the face of the patient, functioning as a facebow [27]. This procedure allowed the dental technician to consider anatomical references (as the bipupillary line) or different anatomical planes (like Camper or Frankfurt) during his work. With this fully digital workflow, it was possible to achieve a predictable and esthetic treatment that matched the patient’s face.

The selection of the most suitable material to restore severe tooth wear cases is still an open issue for discussion [28]. Clinical choices are mostly driven by the medical anamnesis and financial affordability for the patient. Patients who present parafunctional activities may require materials with high mechanical properties, which are fracture- and wear-resistant. In this case, the cause of tooth wear was mostly identified due to the patient’s dietary attitudes, and minor problems were detected regarding the mechanical forces of bruxism. For that reason, resin composite was considered an ideal material for this patient’s therapy, given its high resistance to acidic attack. The resin composite, applied with direct techniques, represented a straightforward and low-cost solution to treating worn dentitions, with satisfying functional and esthetic results [29].

Residual anatomical references (occlusal and interproximal) guided composite shaping and allowed the molding tray to be repositioned precisely, without deformation. Besides the biological advantages of the non-invasive restoration approach, the full-molding technique allowed for the simplification of operative procedures, decreased the number of clinical sessions and, consequently, reduced the cost for the patient. Proper deletion and polishing of resin composite interproximal excesses are the critical steps in this technique, which may result in a possible inaccuracy of the restored anatomy, especially on the lingual side. However, such a risk may be mitigated with a precise definition of the tray limits and improvements in skill made by advancing the learning curve of the operator [21].

The most frequent complications when using resin composite are minor failures such as marginal discoloration or chipping. They usually only require an uncomplicated and rapid repair procedure. In fact, Loomans et al. observed acceptable clinical performance (annual 3% failure) after 3.5 years of tooth wear treated with direct composite [11]. Moreover, the correct use of shade stratification with “the natural layering concept” allow for adequate integration of the restorations with the esthetic of the smile of the patient. They permit more accurate replication of the optical and anatomical characteristics of natural teeth by using two basic masses, dentine and enamel, which mimic natural tooth structure [23].

## 4. Conclusions

Advancements in adhesive dentistry and minimally invasive concepts permit clinicians to take advantage of a wide spectrum of therapeutic options. Most of them result in success if they are well employed and carefully selected in relation to the comprehensive clinical examination. This report shows a worn dentition treated successfully with a combination of direct composites techniques. Hybrid solutions like these can represent valid options, and knowledge of them allows clinicians flexibility in treatment planning.
